# Acute Brucellosis with Splenic Infarcts: A Case Report from a Tertiary Care Hospital in Saudi Arabia

**DOI:** 10.1155/2015/940537

**Published:** 2015-07-13

**Authors:** Mishal Alyousef, Mushira Enani, Mohammad Elkhatim

**Affiliations:** Academic and Training Affairs, King Fahad Medical City, P.O. Box 59046, Riyadh 11525, Saudi Arabia

## Abstract

Splenic infarction is an extremely rare and unique presentation of brucellosis. Only few cases have been reported worldwide. We here report a case of a young man, presenting with acute onset of fever, left hypochondial pain, and vomiting. Further evaluation revealed multiple splenic infarcts and positive blood culture for brucellosis despite negative transesophageal echocardiography for endocarditis. Significant improvement in clinical symptoms and splenic lesions was achieved after six weeks of combination therapy against brucellosis.

## 1. Background

Brucellosis is the most common zoonotic infection worldwide [1]. It is estimated that 500,000 brucellosis cases are reported annually [2]. Brucellosis has a profound public health and economic burden, especially in the Middle East and Mediterranean countries [3]. Brucellosis is hyperendemic in Saudi Arabia with an incidence of 40 cases per 100,000 persons per year [4]. It is caused by gram-negative coccobacilli transmitted to humans via unpasteurized milk, cheese, and fluids of infected animals like cattle, goats, and sheep. It has a wide range of nonspecific clinical manifestations and complications [5, 6]. However, vascular complications are rarely reported in endemic areas. Herein, we report a case of multiple splenic infarcts associated with acute brucellosis.

## 2. Case Presentation

A 17-year-old boy, medically free, with history of unpasteurized milk ingestion, living in Shaqra (a village 200 km far from Riyadh, the capital of Saudi Arabia), presented to our emergency department with fever, vomiting, and malaise for 2 weeks followed by 5-day history of left hypochondrial pain. He lost 9 kgs in the preceding two months. He had no history of trauma or surgery. He was admitted to a peripheral hospital where he received a course of antibiotics with no improvement. On clinical examination, he looked ill, in pain with a temperature of 38.4 degree Celsius; pulse rate of 116 beats per minutes; blood pressure 111/68 mmHg; and respiratory rate of 21 breaths per minute. Abdominal examination revealed moderate tenderness in the left hypochondrium, no organomegaly on palpation and normal bowel sounds.

**Figure 1:**
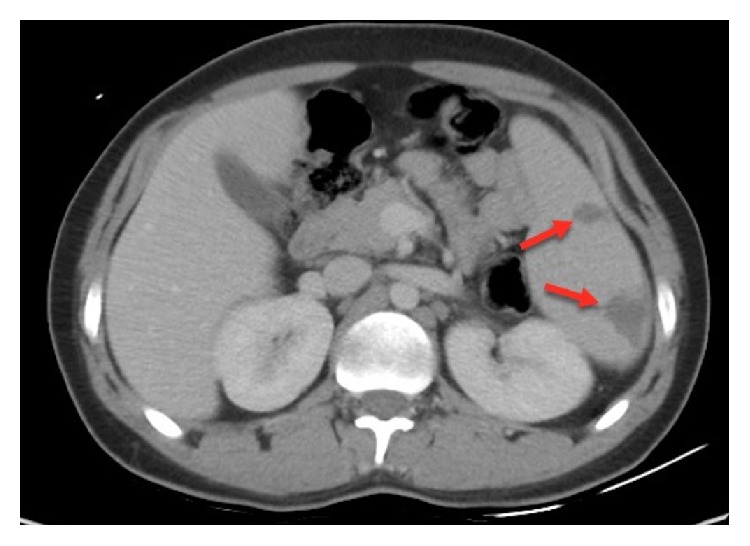
Abdomen CT without contrast showing multiple hypodense splenic lesions.

*Laboratory investigation showed* white blood cell count 4.12 × 10^9^/L; hemoglobin 11.80 g/dL; platelet count 364 × 10^9^/L; ESR 61 mm/H; CRP 66.5 mg/L; amylase 509 U/L; lipase 471 U/L; alanine transaminase 45 U/I; aspartate transaminase 32 U/I; alkaline phosphatase 162 U/I; gamma GT 86 U/I; total bilirubin 0.2 mg/dL; albumin 39 g/L; creatinine 0.5 mg/dL; urea 3.2 mmol/L; and lactate dehydrogenase, 303 U/I. Chest X-ray was normal. Electrocardiogram showed sinus tachycardia. Abdominal X-ray showed no evidence of intestinal obstruction. Computed tomography of abdomen showed multiple irregular peripheral hypodense splenic lesions suggestive of splenic infarcts and borderline splenomegaly, with no evidence of acute pancreatitis and patent splenic veins with no thrombosis (Figure 1). Blood culture grew* Brucella* species after 88 hours of incubation.* Brucella* serology using enzyme-linked immunosorbent assay was positive for IgM. Transesophageal echocardiography was negative. Lupus anticoagulant and anti-cardiolipin antibodies were negative. Proteins C and S were negative. JAK2 mutation was negative.

*Treatment of brucellosis with* rifampicin 600 mg daily (for 6 weeks), doxycycline 100 mg twice daily (for 6 weeks), and gentamycin 300 mg daily (for 7 days) was given to him.

**Figure 2:**
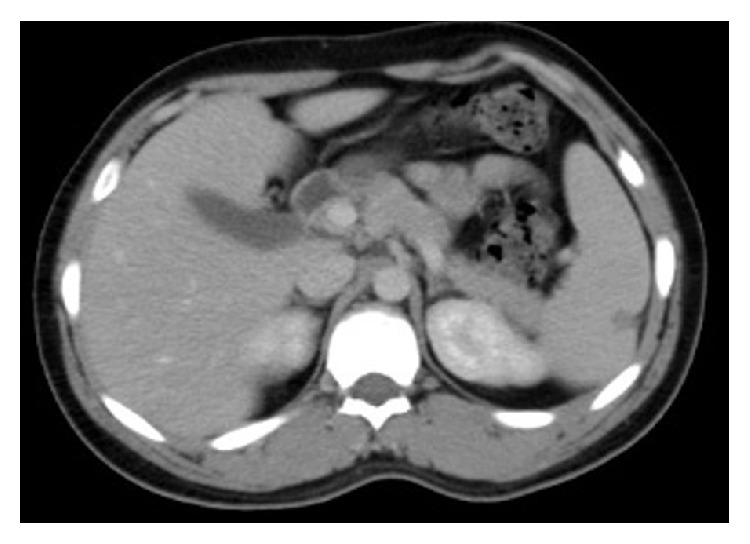
Repeated CT abdomen showing marked regression of the largest lesion while disappearance of others.

On the 8th day of admission, fever and abdominal pain resolved. High amylase and lipase were normalized and the patient was discharged home in good condition. After 3-month follow-up in outpatient clinic, the patient remained well with significant improvement of splenic infarcts on computed tomography (Figure 2).

## 3. Discussion

Vascular complications of brucellosis like deep venous thrombosis, infective endocarditis, and atrial aneurysm formation have been rarely reported [7–9]. Splenic infarction is also a rare complication of brucellosis. To the best of our knowledge, no case of splenic infarction associated with brucellosis has been reported in Saudi Arabia before. Moreover, there are 5 previously reported similar cases in Spain, Italy, Korea, Tunisia, and Turkey [10–14].

The exact pathogenesis of this complication is not clear yet; however, different mechanisms were proposed such as damage to the adjacent tissue during an infectious process, septic embolism, endophlebitis, direct endothelial damage, induction of inflammation, and hypercoagulable state [6, 15].

In our patient, the proinflammatory response induction caused by direct endothelial damage seems to be the most favorable explanation as cardiac and hypercoagulable studies were negative [16].* Brucella* species leading to vascular complications have been identified as* B. melitensis*,* B. abortus*, and* B. suis* [17].* B. melitensis* is most commonly encountered in Saudi Arabia [18].

Acute pancreatitis is also a rare complication of brucellosis; only few cases were reported [19, 20]. However, in our case, acute pancreatitis was the first provisional diagnosis but it was promptly excluded by CT abdomen as it revealed hypodense splenic lesions and borderline splenomegaly with no evidence of acute pancreatitis. In our patient,* Brucella* infection was highly suspected, especially with history of prolonged fever, endemicity of brucellosis in Saudi Arabia, and presence of risk factors; it was confirmed by isolation of* Brucella* species in blood culture.

Symptoms of splenic infarction are nonspecific and may include left hypochondial pain, fever, and vomiting. CT abdomen without contrast is the best imaging modality for detecting splenic infarctions [21, 22].

Our patient was managed conservatively and by commencing triple anti-*Brucella* therapy. An observational study reported that higher frequency of clearance of* Brucella* DNA from blood can be achieved with triple therapy [23].

Anticoagulation administration in splenic infarction is still in debate [24]. However, better results were reported by Cappell and his colleagues only in the presence of antiphospholipid antibody [25].

Thrombocytopenia is a prominent laboratory finding in brucellosis. To our knowledge, thrombocytopenia was reported in the previous cases of splenic infarctions associated with brucellosis. In our case, normal platelet count was observed; therefore bone marrow biopsy was not considered.

## 4. Conclusion

Acute brucellosis should be in the differential diagnosis of patients with splenic infarctions especially in endemic areas. Excluding infective endocarditis is important to plan the duration of therapy and subsequent management.
